# Primary Prevention and Health Promotion Among Refugee Women in Greek Accommodation Facilities

**DOI:** 10.3390/healthcare14040546

**Published:** 2026-02-22

**Authors:** Giannoula Kyrkou, Panagiota Kassiou, Elina Christiana Alimonaki, Maria Iliadou, Victoria Vivilaki, Artemisia Kokkinari, Anna Deltsidou, Angeliki Sarella, Nikoleta Tsinisizeli, Anastasia Bothou

**Affiliations:** 1 Department of Midwifery, School of Health and Care Sciences, University of West Attica, 12243 Athens, Greece; giotakas@hotmail.com (P.K.); e.alimonaki@yahoo.com (E.C.A.); miliad@uniwa.gr (M.I.); vvivilaki@uniwa.gr (V.V.); akokkinari@uniwa.gr (A.K.); adeltsidou@uniwa.gr (A.D.); asare@uniwa.gr (A.S.); nikoletatsinisizeli@gmail.com (N.T.); abothou@uniwa.gr (A.B.); 2 Neonatal Intensive Care Unit, General Hospital of Nikaia “Agios Panteleimon”, 18454 Nikea, Greece

**Keywords:** refugee women, primary prevention, health promotion, healthcare access, cultural barriers, women’s health

## Abstract

**Background/Objectives:** Greece has been a major host country for refugee populations, operating under conditions of limited resources and strained healthcare services. Refugee women residing in accommodation facilities face barriers to accessing primary prevention and health promotion services, including limited health literacy and cultural and linguistic challenges. This study aimed to assess the level of primary prevention and health promotion among refugee women living in accommodation facilities in Greece and to identify their health needs and barriers to accessing healthcare services. **Methods:** A quantitative cross-sectional study was conducted among 150 adult refugee women residing in the Malakasa accommodation facility in Greece. Participants voluntarily agreed to take part in the study. Data were collected between December 2024 and March 2025 using a structured questionnaire assessing sociodemographic characteristics, primary prevention, health promotion, and barriers to healthcare access. Descriptive statistical analysis was performed. The study was approved by the relevant ethics committee, and informed consent was obtained from all participants. **Results:** The study included 150 refugee women, primarily young adults with low educational attainment. Familiarity with primary prevention was reported as moderate or lower by the majority of participants, with only 24% indicating high or excellent familiarity, while familiarity with health promotion was even lower (8%). Participation in preventive practices varied, with 42.7% reporting frequent health check-ups; however, uptake of key preventive behaviors remained limited, including vaccination (30%) and adoption of a healthy diet (32.7%). During their stay in Greece, 97.3% participated in regular health check-ups, 32.7% adopted a healthy diet and 30% were vaccinated. Cardiovascular and gynecological conditions were the most frequently reported health problems (76.7% and 73.3%, respectively). The most prominent barrier to healthcare access was long distance from health facilities (97.3%), followed by lack of information or health education (24.7%). **Conclusions:** The study identified low levels of preventive health knowledge and limited uptake of key preventive practices among refugee women, alongside persistent barriers to healthcare access, underscoring the need for targeted and culturally sensitive health promotion interventions.

## 1. Introduction

Since 2015, Greece has been facing a steady influx of refugees as a result of ongoing wars, armed conflicts, and economic problems in Syria, Iraq, Afghanistan, and some African countries. Thousands of people have crossed the border from Turkey to the islands of the Eastern Aegean and the Evros region, making the country a key gateway to Europe [1]. Greece has been called upon to deal with a major humanitarian crisis, with limited resources and inadequate infrastructure, while living conditions in the hotspots have often been the subject of international criticism [2]. Despite intense pressure and social tensions in areas such as Lesbos and Chios, Greek society has largely shown high levels of solidarity [3].

Refugees arriving in the country often face serious health problems, which are due both to dangerous, long, and arduous journeys and to the conditions in reception facilities. Injuries, infections, undiagnosed or untreated chronic diseases, and aggravating factors such as malnutrition and inadequate vaccination programs have been reported [4,5]. Furthermore, overcrowding in reception facilities further contributes to the spread of communicable diseases and the deterioration of hygiene conditions [6,7]. Refugee women face additional challenges, particularly in relation to sexual and reproductive health. Access to preventive gynecological examinations, family planning services, and prenatal and perinatal care is often limited or sporadic [7,8]. Although refugees have the right to access public health services, a number of barriers prevent them from doing so, such as language and cultural differences, a lack of interpretation services, and inadequate health information [6,9,10]. In this context, understanding how refugee women residing in reception facilities perceive and engage with primary prevention and health promotion becomes particularly important. For the purposes of this study, the term “refugee” is used in a broad, operational sense to describe women residing in reception facilities in Greece who have experienced forced international displacement. This includes asylum seekers and individuals whose legal status has not yet been formally recognized.

Primary prevention and health promotion are key to improving the health of refugee women and reducing the health inequalities they face. Providing accessible and culturally appropriate health education, strengthening health literacy, and developing community support networks are key elements in empowering women, ensuring their equal treatment, and promoting their well-being [11,12].

Given these challenges, it is crucial to explore refugee women’s knowledge, attitudes, beliefs, and practices regarding prevention and health promotion. Understanding the factors that influence access to health services and participation in preventive actions can guide the development of targeted, culturally sensitive, and feasible interventions. This study aims to fill this research gap by providing up-to-date information on the needs and priorities of refugee women living in reception facilities in Greece. By examining multiple dimensions of primary prevention and health promotion, knowledge, practices, access barriers, and perceived needs, this study provides timely and context-specific evidence that has not been sufficiently explored in previous national research. Data on primary prevention and health promotion among refugee women living in reception facilities in Greece remain limited. Existing studies have mainly focused on access to care, maternal health, or communicable diseases, while less attention has been paid to preventive knowledge, practices, and perceived barriers within accommodation settings. The present study addresses this gap by focusing on women residing in a large reception facility and examining multiple dimensions of prevention-related behavior.

### Aim and Objectives

This study aimed to examine knowledge, attitudes, and practices related to primary prevention and health promotion among refugee women residing in a reception facility in Greece.

Specifically, the objectives were to:

- assess participants’ level of knowledge regarding primary prevention and health promotion;
- explore preventive practices and previous engagement in screening services;
- identify perceived barriers to accessing preventive health services;
- examine associations between demographic characteristics and prevention-related behaviors.

## 2. Materials and Methods

### 2.1. Study Design and Setting

This study employed a quantitative, descriptive, cross-sectional design to assess primary prevention and health promotion among refugee women and was reported in accordance with the STROBE (Strengthening the Reporting of Observational Studies in Epidemiology) guidelines. Data were collected at the Malakasa Reception Facility in Greece, which hosts approximately 600 residents, including men, women, and children. At the time of data collection, approximately 250 adult women were residing in the facility. The study included approximately 60% of the eligible adult female population, representing a high level of coverage for this hard-to-reach population. A non-probability convenience sampling approach was used, with voluntary participation among all eligible adult women residing in the facility during the data collection period. The majority of residents are men, and women constitute a smaller subgroup, reflecting the demographic composition of the facility.

### 2.2. Participants

The study focused on adult women residing at the Malakasa Reception Facility who were in situations of forced international displacement, including asylum seekers and individuals with pending or unclear legal status. Participation was voluntary, and the 150 women who took part represented the adult female population present during the data collection period. This subgroup included women of diverse ages, countries of origin, educational backgrounds, and marital statuses, reflecting the heterogeneity of female residents. Participants were recruited using convenience sampling, based on availability and willingness to participate. Although convenience sampling was used, the high participation rate enhances the descriptive validity of the findings within this specific setting.

*Inclusion criteria:*

Participants were eligible if they met all of the following criteria:

Female refugees aged 18 years or older.

Residing in the Malakasa Accommodation Facility for a minimum of three (3) months.

Ability to communicate in Greek, English, Persian, or Arabic (directly or with the help of interpreters).

Provided written informed consent to participate in the study.

Physically and mentally capable of completing the questionnaire.

*Exclusion criteria:*

Participants were excluded if they met any of the following:

Refusal or inability to provide informed consent.

Severe mental or physical illness prevents participation.

Currently participating in another study on health promotion or prevention within the facility (to avoid bias).

Inability to understand the translated questionnaire, even with interpreter assistance.

### 2.3. Study Tool

A structured questionnaire was developed specifically for the purposes of this study. It included sections on: Demographic characteristics, Access to healthcare services, Health education, Perceptions of health before and after arrival in Greece.

The questionnaire was translated from Greek to English, Persian and Arabic by intercultural mediators and interpreters. It was then back-translated into Greek to ensure clarity and semantic accuracy. The translation and back-translation procedures were conducted by bilingual experts to ensure semantic equivalence and cultural relevance of the instruments. A pilot test was conducted with 10 refugee women through face-to-face interviews. Feedback from this process was used to improve the questionnaire.

To assess the stability and clarity of the questionnaire, approximately 20–25 women completed the questionnaire twice over a one-month period. The final version was an anonymous, self-administered, closed-ended questionnaire.

While no formal psychometric validation was conducted, these procedures contributed to ensuring the clarity, relevance, and cultural sensitivity of the instrument.

### 2.4. Data Collection

Data collection took place between December 2024 and March 2025. Before participating in the study, all refugee women were fully informed about the purpose, methodology, and objectives of the study and provided written informed consent. The questionnaires were anonymous in order to ensure confidentiality and protection of personal data.

To enhance cultural and linguistic accessibility, intercultural mediators and professional interpreters were utilized, thus ensuring accurate understanding of the questions and correct completion of the questionnaires. Participation was completely voluntary, without any form of pressure or compensation.

The process of distributing and completing the questionnaires at the Malakasa Hospitality Facility was carried out following official approval by the Ethics Committee of the University of West Attica (76267—23 September 2024).

### 2.5. Statistical Analysis

The distribution of quantitative variables was assessed using the Kolmogorov–Smirnov test for normality. Variables following a normal distribution were described using means (M) and standard deviations (SD), whereas for non-normally distributed variables, medians (Mdn) and interquartile ranges (IQR) were additionally reported.

Absolute frequencies (N) and relative frequencies (%) were used for the description of categorical variables. For comparisons of quantitative variables across more than two groups, the non-parametric Kruskal–Wallis test was applied. For the comparison of proportions, the Pearson’s χ^2^ test or Fisher’s exact test was used, where appropriate.

All tests were two-tailed, and statistical significance was set at *p* < 0.05.

## 3. Results

The results are presented according to the study objectives, focusing on participants’ knowledge, practices, barriers to access, and perceived needs regarding primary prevention and health promotion.

### 3.1. Sample Characteristics

The study included 150 women. Most participants were aged 18–34 years, with smaller proportions in older age groups. Participants originated primarily from Afghanistan, Syria, and Iraq, with additional representation from several African countries. The majority were married and had children (median: 3, IQR: 1–4). Educational attainment was generally low, with most women having either no formal education or only primary schooling. Most participants were engaged in household duties both prior to arrival and at the time of the study (Table 1).

### 3.2. Awareness and Knowledge on Primary Prevention and Health Promotion

Participants demonstrated generally low-to-moderate familiarity with primary prevention, while awareness of health promotion concepts was substantially lower. Only a small proportion reported high familiarity levels. Health professionals were the most frequently reported source of health information, followed by digital media and family or social networks (Table 2).

Overall, the findings indicate low-to-moderate levels of familiarity with primary prevention and particularly limited awareness of health promotion activities among participants.

### 3.3. Preventive Practices and Screening Participation

Most women reported participating in health examinations at least occasionally. Basic laboratory testing was almost universal, and high participation was also observed for gynecological and dental check-ups. Among women who underwent gynecological evaluation, ultrasound examination was most common, followed by vaginal culture and Pap testing.

However, retrospective reporting showed that preventive screening participation prior to arrival in Greece had been limited, with most participants reporting no previous mammography or Pap testing before migration (Table 3).

Despite relatively high participation in medical examinations within the current context, engagement in organized preventive screening practices before arrival in Greece appeared substantially lower.

### 3.4. Barriers to Accessing Primary Prevention and Health Promotion Services

Participants reported substantial barriers to accessing healthcare both in their countries of origin and in Greece. In their previous communities, the most frequently reported barriers were poor healthcare infrastructure, lack of health information or education, and service costs. In their current setting in Greece, distance from healthcare facilities emerged as the dominant barrier, followed by limited access to information.

**Figure 1:**
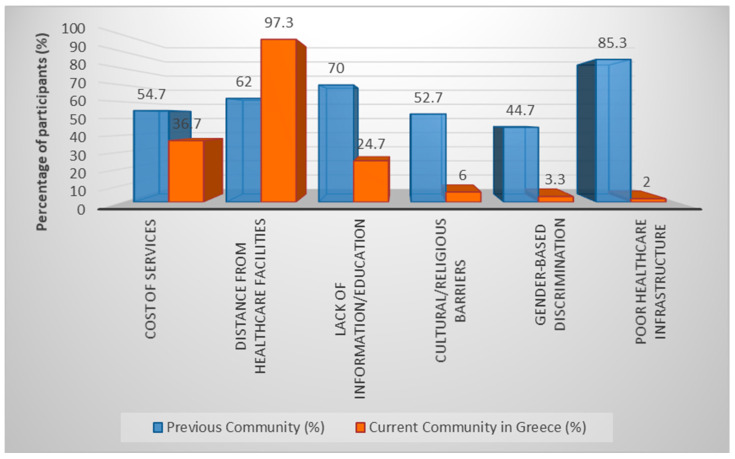
Barriers to Accessing Primary Prevention and Health Promotion Services.

**Figure 2:**
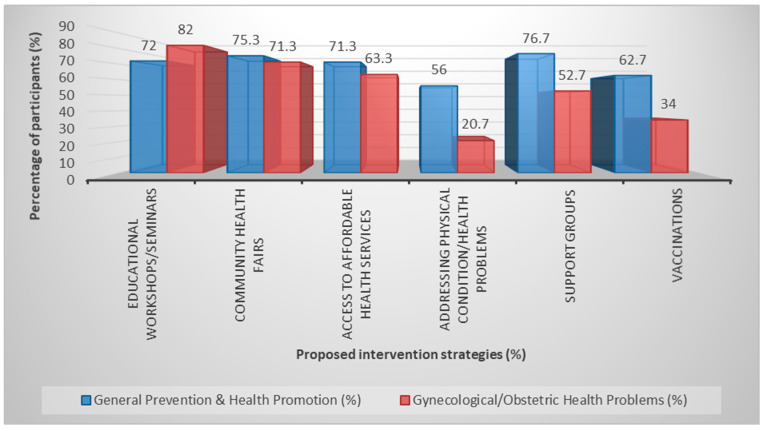
Ways to improve primary prevention and health promotion.

Participants identified several potential strategies for improving prevention and health promotion services. The most frequently selected approaches included support groups, community health initiatives, and educational workshops. Similar strategies were proposed for improving gynecological healthcare access (Figure 1 and Figure 2).

These findings help identify priority areas for intervention, particularly improving geographical accessibility to healthcare services and strengthening health information and education programs for refugee women.

### 3.5. Differences in Familiarity with Primary Prevention and Health Promotion Across Demographic Groups

Statistically significant differences in familiarity with primary prevention and health promotion were observed across country of origin, age groups, and educational levels. Higher familiarity scores were recorded among participants from Afghanistan and African countries compared with those from Iraq. Differences were also observed between younger and older participants. Familiarity scores increased across successive educational categories, with the lowest values recorded among women without formal education (Table 4).

Overall, demographic factors were significantly associated with knowledge and awareness of prevention concepts.

### 3.6. Frequency of Health Screenings and Perceived Importance of Prevention and Health Promotion

Participation in health screenings also differed across demographic groups. Women from Afghanistan and African countries reported higher screening participation compared with women from Iraq. Higher participation was additionally observed among women with secondary or higher education.

Perceived importance of prevention and health promotion followed similar patterns, with higher ratings reported among younger participants and those with higher educational attainment (Table 5).

Overall, demographic characteristics were associated with both participation in preventive practices and the perceived importance of prevention.

## 4. Discussion

Refugee women in the present study demonstrated limited familiarity with the concepts of primary prevention and health promotion, despite expressing willingness to participate in relevant activities when opportunities were available. Access to health services was hindered by structural barriers, mainly distance from facilities and lack of accurate information, while age, country of origin, and particularly educational level appeared to influence levels of knowledge and participation in preventive examinations. From a theoretical perspective, the results can be interpreted within the framework of health literacy and the social determinants of health, suggesting that limited engagement in preventive practices reflects not only individual knowledge gaps but also broader structural constraints related to education, language proficiency, legal status, and access to information. It should also be noted that the study population was legally heterogeneous, including women at different stages of the asylum process, a factor that may have influenced access to healthcare and preventive practices. Although the sample was not statistically representative, the descriptive findings provide valuable exploratory insights into prevention-related knowledge, attitudes, and practices within a hard-to-reach and socially vulnerable population and may inform future large-scale and longitudinal research.

Although this study is the first to focus exclusively on primary prevention and health promotion among refugee women residing in the Malakasa reception facility, the findings are consistent with evidence from other studies conducted in Greece, particularly regarding the influence of linguistic, cultural, economic, and social factors on access to healthcare. Research conducted at the Filippiada Reception Center reported significant barriers to accessing obstetric services, including distance, travel costs, administrative bureaucracy, and lack of interpretation [13], while the ORAMMA project highlighted the role of socioeconomic and legal status in shaping access to perinatal care. In line with this evidence, the present study confirms that educational level and age are key factors influencing refugee women’s knowledge and attitudes toward primary prevention, reinforcing the view that access to healthcare is not determined solely by logistical availability but is closely linked to social integration processes and broader social inequalities [14].

Evidence from refugee camps on Lesvos has highlighted the psychosocial consequences of living in restrictive conditions, including increased mental health burden and reduced ability to utilize health services [8]. Although the present study focused on prevention rather than mental health, the findings similarly indicate limited access to services and low familiarity with health promotion, which may be linked to adverse living conditions within reception facilities. In addition, during the COVID-19 pandemic, the Voices of Immigrant Women program documented increased vulnerability and marginalization due to service disruption and institutional barriers [15]. While the present study did not examine the pandemic period, the findings suggest that refugee women continue to experience multi-level exclusions in accessing preventive healthcare.

Overall, the present study contributes new evidence by identifying education and age as important factors associated with knowledge and participation in preventive examinations, a relationship that has not been sufficiently highlighted in Greek studies to date. It should be noted, however, that age and educational level are closely related variables in this population, and a degree of collinearity may exist between them. Consequently, the observed associations should be interpreted with caution, as the independent effect of each factor could not be fully disentangled within the descriptive analytical framework of the study.

The findings of the present study are consistent with international research indicating low levels of knowledge regarding primary prevention and health promotion among refugee and immigrant women, particularly in relation to preventive gynecological screening (e.g., Pap test, mammography) [16,17]. In our sample, 67% of participants reported unfamiliarity with the concept of health promotion, and a large proportion indicated no prior experience with preventive examinations before arriving in Greece. Similar patterns have been reported among refugee women in countries such as Turkey and Jordan, where low health literacy levels—reflected, for example, in mean EMAHL13 scores of 17.7 among female refugees—have been attributed to cultural and language barriers and the limited availability of preventive health programs in countries of origin. Together, these findings suggest that limited engagement in preventive care reflects systemic barriers rather than individual unwillingness.

Similar patterns have been reported in large international datasets and global health reports, which consistently show that refugee and migrant women experience lower health literacy, reduced familiarity with preventive services, and multiple structural barriers to accessing healthcare. Large-scale analyses from international organizations such as the World Health Organization and the United Nations High Commissioner for Refugees highlight the combined influence of education, socioeconomic vulnerability, and service accessibility on preventive health participation among displaced populations [18,19]. These broader findings support the trends observed in the present study.

International evidence consistently identifies language barriers, lack of interpretation, bureaucracy, and limited access to information as major obstacles to healthcare access for refugee and immigrant women [20,21,22]. Similar barriers were observed in the present study, particularly related to distance from services and insufficient information, reinforcing the shared experience of vulnerable populations across different reception contexts. In addition, international literature highlights the role of educational level in shaping familiarity with and participation in preventive health actions [16,23]. Consistent with these findings, educational level emerged as a key determinant in the present study, with women who had secondary or tertiary education demonstrating greater familiarity with prevention and more frequent participation in preventive examinations. Younger age was also associated with a more positive attitude toward prevention. Although these associations were consistently observed, their independent effects could not be fully disentangled within the descriptive analytical framework of the study, indicating the need for multivariate analyses in future research.

Social perceptions of women’s health and women’s position within the family and broader social context have been shown to substantially influence health-related behaviors. Although cultural factors were not directly explored through qualitative methods in the present study, several findings indirectly reflect culturally mediated patterns, including limited prior exposure to preventive services in countries of origin, differences in familiarity with screening practices, and variations observed by country of origin. These observations are consistent with previous research indicating that social attitudes, shame, religious norms, and cultural beliefs may negatively affect participation in preventive examinations such as Pap tests and mammography [17,21,24]. In this context, the observed differences between women from different regions (e.g., greater familiarity among women from Africa and Afghanistan compared to Iraq) underscore the relevance of culture and support the need for culturally responsive approaches to improve access to and engagement in preventive care.

International evidence indicates that living in refugee camps is associated with limited access to health services and increased psychosocial stress, with refugees facing additional functional and psychosocial barriers to healthcare utilization, including avoidance of preventive services due to trauma or fear [24]. Although the present study focused on prevention rather than mental health, the findings suggest that residence in the Malakasa reception facility similarly affects women’s ability to utilize health services, reinforcing international concerns regarding health inequities in camp settings. In this context, evidence from systematic reviews shows that culturally and linguistically appropriate, multi-component interventions—such as group-based approaches, use of interpreters, multimedia tools, and community participation—can significantly increase participation in preventive screening [23]. The present findings are consistent with this evidence and further support the need for culturally sensitive and context-specific interventions within reception facilities.

Regarding the findings on the participation of refugee women in routine gynecological check-ups, a study based on data from the Canadian Community Health Survey (2012) found that 28.9% of immigrant women were under-encouraged to have a Pap test—compared to 18.6% of native-born Canadians. Immigrant status was associated with a 32% increased relative risk of under-encouragement, regardless of length of stay in the country [20]. Importantly, the high participation rates observed in the present study should be interpreted in relation to participants’ previously limited access to preventive healthcare. Approximately 70% of women reported never having undergone preventive examinations prior to arrival in Greece. Following entry into the reception system, structured intake procedures, routine health assessments, facilitated access to services, and systematic encouragement by healthcare professionals appear to have substantially increased participation in preventive examinations. These findings highlight pre-existing barriers to healthcare access and health information in countries of origin. In a subsequent 2017 study in Ontario, compliance rates (Pap test every three years) were lower (71.3%) among immigrant women compared to non-immigrant women (75.4%). Younger ethnic groups, such as South Asian women, had lower compliance, regardless of educational level and access to a family doctor [25]. The effect of immigration status, origin, and educational level on access to and participation in preventive examinations is consistent with the findings of the present study. A larger study of Kosovo refugees resettled in Canada (1999) found very low rates of screening recall: only 5.3% of women over 50 had had a mammogram and 34.1% had had a Pap test. At the same time, a high rate of Post-Traumatic Stress Disorder (PTSD) (25.9%) was recorded and issues such as lack of language support, difficulty finding a family doctor, and cultural differences in health behavior emerged [26]. Also, an extensive review of immigrant and refugee women in European Union countries showed lower participation in Pap tests compared to native women. The main barriers were lack of information, language problems, shame, emotional resistance, negative religious and cultural values, fears and traumatic experiences. Solutions suggested by the researchers were information and materials in understandable languages, female health professionals, provider education and addressing value conflicts [21]. In Belgium, Italy, Malta, Portugal, and Spain, immigrants reported lower access to both mammography (−46%) and Pap tests (−5%) compared to native women. Education appeared as a protective factor [27].

Regarding standard breast cancer screening and mammography, among 18 European studies, immigrants had an OR = 0.54 compared with natives for Pap test compliance, with particularly low performance in women from North Africa and sub-Saharan Africa (OR = 0.47 and OR = 0.35), showing significant heterogeneity by country of origin [28]. Despite the high mammogram rate (~95%) among Swedish-born women, only about 60% of immigrant women participated. The main exclusion factors were: ignorance, language difficulty, cultural refusal to be screened except for symptoms, fear of male examiners, and a feeling that health is a matter of “divine will” [29]. Taken together, the findings highlight how individual characteristics, structural barriers, and culturally shaped experiences interact to influence preventive health behaviors among refugee women living in reception facilities.

### Strengths and Limitations

This study fills an important gap in the literature, as it is the first to focus on primary prevention and health promotion among refugee women in the Malakasa reception facility. The methodological approach was reinforced by the use of tools and interpreters adapted to the cultural and linguistic context, thus ensuring clarity and broader participation. In addition, the analysis of key demographic factors, such as origin, educational level, and age, provided valuable information for understanding prevention behaviors and attitudes.

Nevertheless, several limitations should be acknowledged. The use of non-random, convenience sampling limits the statistical representativeness of the sample and may have introduced selection bias, as participation relied on voluntary response. The cross-sectional design precludes causal interpretation of the findings, and the descriptive analytical approach did not allow for the assessment of independent effects between variables or the use of multivariate analyses. In addition, although the questionnaire was culturally adapted, the absence of full psychometric validation may affect measurement reliability. Finally, reliance on self-reported data may have introduced reporting or recall bias, and the relatively short data collection period did not permit assessment of long-term outcomes while the limited data collection period did not allow for the recording of long-term data and results.

## 5. Conclusions

The study reveals the limited familiarity of refugee women in Malakasa with primary prevention and health promotion, despite their willingness to participate in related actions. The main barriers observed are geographical distance from health care facilities and insufficient access to reliable information. In addition, factors such as education and age emerged as important determinants of knowledge and participation in preventive screenings, highlighting the need for personalized interventions that take into account their demographic and sociocultural diversity.

The results highlight the urgent need for culturally sensitive and accessible health care services. Community initiatives, such as support groups, health fairs and educational workshops, would help bridge the access gap and promote social inclusion. Addressing these barriers is not simply an individual health issue, but a public health priority, as the health of refugee women is inextricably linked to the overall well-being and inclusion of their communities.

To strengthen the evidence base and inform policy and practice, future research should utilize longitudinal and mixed methodological approaches, ensure psychometric validation of instruments, and scale up to multiple refugee-hosting settings in Greece and Europe.

## Figures and Tables

**Table 1:** Demographic characteristics of the participants.

		N	%
**Age**	18–24	42	28.0
	25–34	40	26.7
	35–44	31	20.7
	45–54	19	12.7
	55+	18	12.0
**Country of Origin**	Afghanistan	47	31.3
	Syria	33	22.0
	Iraq	24	16.0
	Ghana	5	3.3
	Guinea	9	6.0
	Eritrea	14	9.3
	Nigeria	6	4.0
	Kenya	4	2.7
	Sudan	8	5.3
**Marital Status**	Single	25	16.7
	Married	96	64.0
	Divorced	11	7.3
	Widowed	18	12.0
**Number of Children,** Mean (SD), Median (IQR)		2.8 (2.0)	3.0 (1–4)
**Educational Level**	No formal education (illiterate)	51	34.0
	Primary education	52	34.7
	Secondary education	37	24.7
	Higher education (college/university)	10	6.7
**Previous Employment Status**	Student	14	9.3
	Employed	33	22.0
	Unemployed	7	4.7
	Household duties	95	63.3
	Other	1	0.7
**Current Employment Status**	Student	5	3.3
	Employed	25	16.7
	Unemployed	29	19.3
	Household duties	91	60.7

N = number of respondents.

**Table 2:** Familiarity with primary prevention and health promotion.

		N	%
**Familiarity with primary prevention**	Not at all familiar	28	18.7
	Slightly familiar	37	24.7
	Moderately familiar	49	32.7
	Very familiar	27	18.0
	Extremely familiar	9	6.0
**Familiarity with health promotion**	Not at all familiar	67	44.7
	Slightly familiar	32	21.3
	Moderately familiar	39	26.0
	Very familiar	9	6.0
	Extremely familiar	3	2.0
**Source of information**	Health professionals	116	77.3
	Internet/social networks	91	60.7
	Television/radio	47	31.3
	Community health workers	58	38.7
	Family/friends	90	60.0

N = number of respondents.

**Table 3:** Participation in preventive health screenings.

		N	%
**Frequency of health screening participation**	Never	7	4.7
	Rarely	46	30.7
	Often	64	42.7
	Annually	21	14.0
	Whenever indicated	12	8.0
Types of screening tests performed *	Blood test	145	96.7
	Imaging test	85	56.7
	Cardiology check-up	62	41.3
	Dental check-up	114	76.0
	Gynecological check-up	129	86.0
Gynecological examinations performed *	Ultrasound examination	110	85.3
	Hormonal test	43	33.3
	Pap smear test	64	49.6
	Vaginal swab culture	79	61.2
	Mammogram	43	33.3
**Mammography before arrival in Greece**	1 year ago	20	13.3
	2 years ago	14	9.3
	3–4 years ago	9	6.0
	Never	107	71.3
**Pap smear before arrival in Greece**	1 year ago	47	31.3
	2 years ago	17	11.3
	3–4 years ago	2	1.3
	Never	84	56.0

N = number of respondents, * Multiple responses allowed.

**Table 4:** Familiarity with Primary Prevention and Health Promotion by Country of Origin, Age, and Educational Level.

		Primary Prevention Mean (SD)	*p* Value	Health Promotion Mean (SD)	*p* Value
**Country of Origin**	Afghanistan	2.8 (0.9)	<0.001 ^++^	2.1 (0.9)	<0.001 ^++^
	Syria	2.5 (1.1)		1.6 (0.9)	
	Iraq	1.8 (0.8)		1.4 (0.7)	
	African countries	3.1 (1.3)		2.5 (1.2)	
**Age**	18–24	2.8 (1.1)	<0.001 ^++^	2.2 (1.0)	<0.001 ^++^
	25–34	3.1 (1.1)		2.4 (1.1)	
	35–44	2.8 (1.2)		1.9 (1.0)	
	45+	2.0 (1.0)		1.5 (0.9)	
**Educational Level**	No formal education (illiterate)	1.7 (0.7)	<0.001 ^++^	1.2 (0.5)	<0.001 ^++^
	Primary education	2.8 (0.9)		1.8 (0.8)	
	Secondary education	3.5 (0.8)		2.9 (0.8)	
	Higher education	4.2 (1.0)		3.7 (0.8)	

SD = standard deviation, ^++^ Kruskal–Wallis test.

**Table 5:** Frequency of Participation in Health Screenings and Importance of Primary Prevention and Health Promotion by Country of Origin, Age, and Educational Level.

		Frequency of Participation Mean (SD)	*p* Value	Importance of Prevention & Health Promotion Mean (SD)	*p* Value
**Country of Origin**	Afghanistan	3.0 (0.9)	0.006 ^++^	4.0 (1.1)	0.017 ^++^
	Syria	2.8 (0.8)		3.9 (1.1)	
	Iraq	2.3 (0.8)		3.0 (1.2)	
	African countries	3.2 (1.2)		4.3 (0.8)	
**Age**	18–24	2.8 (1.1)	0.098 ^++^	4.2 (1.0)	<0.001 ^++^
	25–34	3.2 (1.0)		4.3 (0.8)	
	35–44	3.0 (0.9)		3.8 (1.0)	
	45+	2.6 (0.8)		3.2 (1.3)	
**Educational Level**	No formal education (illiterate)	2.5 (0.7)	<0.001 ^++^	3.2 (1.2)	<0.001 ^++^
	Primary education	2.8 (0.8)		4.1 (0.9)	
	Secondary education	3.4 (1.0)		4.6 (0.6)	
	Higher education	4.2 (1.2)		4.7 (0.9)	

SD = standard deviation, ^++^ Kruskal–Wallis test.

## Data Availability

All data generated or analyzed during this study are included in this published article. Additional data may be provided by the corresponding author upon reasonable request.
